# Identification of novel pathogenic variants of *Calpain-3* gene in limb girdle muscular dystrophy R1

**DOI:** 10.1186/s13023-024-03158-1

**Published:** 2024-04-01

**Authors:** Sukanya Banerjee, Bishan Dass Radotra, Manni Luthra-Guptasarma, Manoj K Goyal

**Affiliations:** 1 Department of Histopathology, Post Graduate Institute of Medical Education and Research, 160012 Chandigarh, India; 2 Department of Immunopathology, Post Graduate Institute of Medical Education and Research, 160012 Chandigarh, India; 3 Department of Neurology, Post Graduate Institute of Medical Education and Research, 160012 Chandigarh, India

**Keywords:** Calpain-3, LGMDR1, Immunohistochemistry, Western blotting, Sanger sequencing

## Abstract

**Background:**

Limb Girdle Muscular Dystrophy R1 (LGMDR1) is an autosomal recessive neuromuscular disease caused by mutations in the *calpain-3* (*CAPN3)* gene. As clinical and pathological features may overlap with other types of LGMD, therefore definite molecular diagnosis is required to understand the progression of this debilitating disease. This study aims to identify novel variants of *CAPN3* gene in LGMDR1 patients.

**Results:**

Thirty-four patients with clinical and histopathological features suggestive of LGMD were studied. The muscle biopsy samples were evaluated using Enzyme histochemistry, Immunohistochemistry, followed by Western Blotting and Sanger sequencing. Out of 34 LGMD cases, 13 patients were diagnosed as LGMDR1 by immunoblot analysis, demonstrating reduced or absent calpain-3 protein as compared to controls. Variants of *CAPN3* gene were also found and pathogenicity was predicted using in-silico prediction tools. The *CAPN3* gene variants found in this study, included, two missense variants [*CAPN3*: c.1189T > C, *CAPN3*: c.2338G > C], one insertion-deletion [c.1688delinsTC], one splice site variant [c.2051-1G > T], and one nonsense variant [c.1939G > T; p.Glu647Ter].

**Conclusions:**

We confirmed 6 patients as LGMDR1 (with *CAPN3* variants) from our cohort and calpain-3 protein expression was significantly reduced by immunoblot analysis as compared to control. Besides the previously known variants, our study found two novel variants in *CAPN3* gene by Sanger sequencing-based approach indicating that genetic variants in LGMDR1 patients may help to understand the etiology of the disease and future prognostication.

**Supplementary Information:**

The online version contains supplementary material available at 10.1186/s13023-024-03158-1.

## Introduction

Limb girdle muscular dystrophy (LGMD) refers to a group of rare, highly heterogeneous, autosomal neuromuscular disorders. Currently over thirty subtypes are described, most of which result in proximal muscle weakness or myalgia [1]. LGMD is divided into autosomal dominant and autosomal recessive types based on inheritance pattern [2]. Dominantly inherited LGMD is less common than recessive (5–10% of all LGMD) [3]. More than 40 different genes associated with muscular dystrophy have been identified till date including nine autosomal dominant and 29 autosomal recessive forms (LGMDR1 to LGMDR24) [4]. It has been reported that the most common LGMD subtype is LGMDR1 (26.5–30% of LGMD) [5] which occurs due to a number of variants in *CAPN3* gene [6]. However, the frequency can vary widely within population [7]. Onset of symptoms ranges from 6 months to 14 years [8]. The most common phenotype of LGMDR1 is progressive muscle weakness and atrophy, which is usually accompanied by bilateral scapular winging and Achilles tendon contracture [9]. The clinical phenotype and laboratory data are not specific for LGMDR1 and may overlap with other types of LGMD; thus, the disease can be neither clinically diagnosed with certainty nor investigated at the biochemical level. Slowly progressive LGMDR1 can cause loss of ambulation during childhood and near-normal life expectancy [10]. To our best knowledge, calpain-3 immunoblot is the primary method to diagnose LGMDR1 in clinically suggestive cases before conclusive diagnosis based on immunohistochemistry and molecular genetics [8]. Therefore, the current practice involves demonstration of calpain-3 protein expression by immunoblot analysis followed by genetic analysis of *CAPN3* gene for diagnosis of LGMDR1. The histopathological findings in muscle biopsy of LGMDR1 include muscle necrosis, interstitial macrophage, regeneration, muscle fiber size variation [11–15]. Endomysial inflammation may be observed in an occasional case. The histopathological features are not specific; therefore, diagnosis remains a challenge [12] and ancillary techniques are required.

More than 440 different pathogenic variants of calpain-3 gene have been reported till now, distributed along the whole length of gene product [16]. *CAPN3* gene comprises of 24 exons and is located on chromosome 15q15.2 [17]; it is expressed as a 3.5-kb transcript and encodes a 94 kDa calpain-3 protein [18]. Calpain-3 is a calcium dependent non-lysosomal intracellular cysteine protease comprising of 821 amino acids. It has an N-terminal domain (domain I), proteolytic domain (domain IIa and IIb), a calcium and phospholipid binding C2 domain (domain III), calcium binding domain (domain IV with five EF hand sequences which help to bind with calcium ions) and three unique sequences known as NS1, IS1, IS2 [19]. In addition, calpain-3 protein is involved in muscle membrane repair and maintenance [20] and myogenesis [21]. However, its physiological functions are yet to be studied [22]. As per literature, most frequent type of variants found in *CAPN3* gene is missense variants (approx 60%) among which incidence of heterozygous variants is common [23]. Currently there are no hotspot mutation regions knowns in *CAPN3* gene [24]. Moreover, *CAPN3* variants often represent irregular distribution along the entire length of the gene. However few studies showed that cluster of variants might be present in a subgroup of exons in several populations [11].

Diagnosis of calpainopathy depends mainly on the detection of calpain-3 protein expression. Phenotypes of LGMDR1 are sometimes overlapped with other muscular dystrophies. Demonstration of protein deficiency in muscle tissue by Western blot helps the diagnosis, but protein expression may be normal in some cases. Therefore, demonstration of *CAPN3* gene mutations confirms the diagnosis. In the present study, we investigated calpain-3 protein expression in muscle dystrophy cases suspected to have calpainopathy, for which muscle enzyme histochemistry and immunohistochemistry were performed. We hypothesized that analysis of calpain-3 protein expression would help in the diagnosis. This diagnosis can be further confirmed at genetic level by detection of *CAPN3* gene mutations in all 24 exons that may provide a new insight in diagnosis of LGMDR1 patients.

## Materials and methods

The study was conducted at Post Graduate Institute of Medical Education and Research (PGIMER) Chandigarh. Patients with muscular dystrophy presenting to Neurology department were included. A clinical diagnosis of muscular dystrophy was made on clinical findings. The Creatine Kinase (CK) level and myopathic pattern on EMG were recorded. The demographic and clinical details of LGMD patients including age, sex and clinical records were taken from the Department of Neurology. A total of 34 cases with dystrophic features on histology and suspected of LGMD were enrolled in the study. Muscle biopsy was taken mainly from biceps or deltoid; however, some were obtained from hamstring and vastus lateralis muscles. A vastus lateralis muscle samples free of any muscle disease obtained from the thigh flaps used during craniectomy procedures were taken as control (*n* = 5). Histopathologically, proven LGMD of both sexes were included for this study. Patients with inflammatory myopathy were excluded from the study. An informed consent from all the patients was collected. The ethical clearance was obtained from the institute’s ethics committee (NK/4563/PhD).

### Histopathology

Muscle biopsy tissue sections were processed for Haematoxylin & Eosin (H&E) staining described below. 8 μm of tissue sections were cut and lifted on glass slides. Slides were mounted in DPX after completion of H&E staining.

### Enzyme histochemistry

Muscle biopsy samples were processed and the histochemical stains NADH-TR (Nicotinamide adenine dinucleotide-tetrazolium reductase), SDH (succinic dehydrogenase), COX-SDH (Cytochrome oxidase-succinic dehydrogenase) used on cryosections (8-µm thickness). NADH-TR stain was used to differentiate Type I and Type II fibers, distribution of mitochondria, abnormal mitochondria; SDH to detect fibers with abnormal mitochondria; Cox to detect fibers without activity; COX-SDH to detect fibers without cytochrome-C oxidase activity.

### Immunohistochemistry

IHC was done to exclude other types of dystrophies with various primary antibodies such as anti-dystrophin antibody (#Invitrogen PA5-32388), anti-sarcoglycan alpha antibody (#Novus Biologicals NBP2-67150), anti-sarcoglycan beta antibody (#Novus Biologicals NBP2-54977), anti-sarcoglycan gamma antibody (#Novus Biologicals NBP1-90299), anti-dysferlin antibody (#Abcam 124,684).

### Western blotting of calpain-3 protein

Total protein was extracted from snap-frozen muscle biopsy tissues (10 mg) using lysis buffer (0.125 M Tris, 10% glycerol, 4% SDS; pH 8.0) [25] and supernatant was quantitated by bicinchoninic acid (BCA) assay. Forty microgram (40 µg) protein was subjected to 10% SDS-PAGE (sodium dodecyl sulphate-polyacrylamide gel electrophoresis) and electroblotted by using a semi-dry transfer system. Membrane was blocked with 5% skimmed milk in 1x TBST (Tris Buffered Saline with 0.1% Tween-20) 4°C overnight. Next day, membrane was incubated with primary antibody (Anti-calpain-3, Abcam 103,250) for 2 h at room temperature. Then membrane was incubated with secondary antibody for 1 h at room temperature and then developed with chemiluminescence substrate. After this, the membrane was stripped off and probed with anti-GAPDH antibody as GAPDH was used as a loading control. Results of individual patients were analyzed with control. Here, we used one control per one blot for analysis. Further, analysis of Western blotting was done by using ImageJ software (https://imagej.en.softonic.com/download). We could not repeat the GAPDH analysis with a low exposure because we do not have enough samples to repeat.

### Mutation analysis of *CAPN3* gene by Sanger sequencing

The genomic DNA was isolated from peripheral blood using DNA isolation kit (Qiagen) as per manufacturer’s protocol. The isolated DNA was subjected to PCR amplification with primers for all 24 exons of *CAPN3* gene. PCR was done with 70–200 ng DNA and amplified DNA was analyzed by 2% agarose gel electrophoresis. Amplified PCR products were purified using PCR purification kit (Qiagen) as per manufacturer’s protocol. Purified PCR products then proceeded for DNA sequencing using terminator kits based on Sanger Sequencing. The sequencing data obtained from Sanger Sequencing was visualized using chromatogram viewer FinchTV 1.4 software (https://finchtv.software.informer.com/1.4/) and matched with NCBI reference sequence using Nucleotide blast. Data annotation was done by Ensemble genome browser (https://asia.ensembl.org/index.html). The nomenclature of variants reported in this study is based on transcript NM_000070.3 (*CAPN3*). Variants were also validated in parents as per the availability of samples. In-silico analysis was done for pathogenic and likely pathogenic variants using these prediction tools: PolyPhen-2 (http://genetics.bwh.harvard.edu/pph2/), Provean (http://provean.jcvi.org/index.php), Mutation Taster (http://www.mutationtaster.org/), Human Splice Site Finder. Clinvar database was searched for the variants. The variants found in this study were classified according to ACMG (American College of Medical Genetics and Genomics) guidelines by Richard et al. 2015 [26].

### Statistical analysis

Statistical analysis was performed using GraphPad Prism 10 Software. Data were tested for normal distribution using Shapiro-Wilk test. Data were presented as median. Mann-Whitney test was used for group comparison where data were non-normal. The difference between two groups was considered significant where the P value was < 0.05.

## Results

### Clinical finding

According to Calpainopathy Review in Gene Reviews 2022, calpainopathy patients present some clinical features such as tiptoe walking, difficulty in running, scapular winging, waddling gait, laxity of the abdominal muscles, Achilles tendon shortening, and scoliosis. These patients do not have cardiac involvement or intellectual disability.

Previous study by Piluso G et al., 2005 [16] reported autosomal recessive calpainopathy phenotypes based on the distribution of muscle weakness and age at onset into five categories: (A) Severe LGMD patients had serious weakness and required wheelchair before the age of 35; (B) intermediate LGMD patients had weakness and difficulty in deambulation, but they were able to walk alone beyond the age of 35; (C) mild LGMD patients had symmetrical scapular winging and hyperCKaemia; (D) isolated hyperCKaemia; and (P) was characterized by presymptomatic patients who were too young to be classified by clinical criteria but showed laboratory findings such as increased CK, myopathic biopsy, and others.

In our study, all patients showed mainly proximal limb weakness (proximal > distal), difficulty in climbing upstairs and getting up from sitting position as clinical manifestations. Serum CK level ranged between 472 and 20,000 IU/L. None of the patients had facial or cardiac disease. Scapular winging was observed as one of the major clinical features. Out of 13 LGMDR1 patients, there were 9 males and only 4 females with male: female ratio of 2.25:1. The median age of presentation was 22 with a range of 5–57 years. The Table 1 represents genotype-phenotype correlation of LGMDR1 patients with *CAPN3* variants in different exons.

**Table 1:** Variants in *CAPN3* gene found in LGMDR1 patients, *in-silico* analysis and genotype-phenotype correlation

Genotype-phenotype correlation	Waddling gait; wasting of biceps, triceps and supraspinatus muscle; Calf with EDB (extensor digitorum brevis) muscle and Beevor sign were observed	Waddling gait and scapular winging	Proximal weakness since 2 years	Problems in running since the last 6 years, difficulty in getting up	Difficulty in climbing stairs, getting up from squatting position. Waddling gait, scapular winging and adductor splay sign were observed	Patient needed support during walking, with presence of lordotic posture and was wheelchair bound
ACMG criteria and classification	PM1, PM2, PP2, PP3 (Likely pathogenic)	PM1, PM2, PP2, PP3, PP5 (Pathogenic)	PVS1, PM2, PM3, PP3, PP5 & PM1, PM2, PM3, PP2, PP3, PP5 (Pathogenic)	PVS1, PM2, PP3 (Pathogenic)	PM1, PM2, PP2, PP3, PP5 (Pathogenic)	PVS1, PM2, PP3, PP5 (Pathogenic)
Human Splice Site Finder			Acceptor splice site affected			
Mutation Taster	Disease causing	Disease causing	Disease causing	Disease causing	Disease causing	Disease causing
Provean	del (-5.318)	del (-5.973)	del (-5.973)		del (-5.973)	
PolyPhen	PD (0.929)	PD (1.000)	PD (1.000)		PD (1.000)	
Mutation type	Missense	Missense	Splice site variant & missense	Insertion-deletion	Missense	Nonsense
HGVSp	p.Phe397Leu	p.Asp780His	p.Asp780His	p.Arg490Leufs*87	p.Asp780His	p.Glu647Ter
HGVSc	**c.1189T > C**	c.2338G > C	c.2338G > C & c.2051-1G > T	**c.1688delinsTC**	c.2338G > C	c.1939G > T
Genotype	Homo	Homo	Hetero	Homo	Homo	Homo
Exon	9	22	22 & splice site variant	11	22	17
Gene	*CAPN3*	*CAPN3*	*CAPN3*	*CAPN3*	*CAPN3*	*CAPN3*
Patient (Age/Sex)	1 (19/M)	2 (45/M)	3 (25/F)	4 (26/M)	5 (26/F)	6 (22/M)

Homo- Homozygous, Hetero- Heterozygous, PD- Probably damaging, del- Deleterious, Variants reported based on transcripts NM_000070.3 (*CAPN3*)]. Novel variants found in this study are highlighted in bold. All variants are classified as per guidelines provided by American College of Medical Genetics and Genomics (ACMG) [26]. We have incorporated the ACMG criteria for all *CAPN3* variants in Table 1

### Pathological finding

**Fig. 1:**
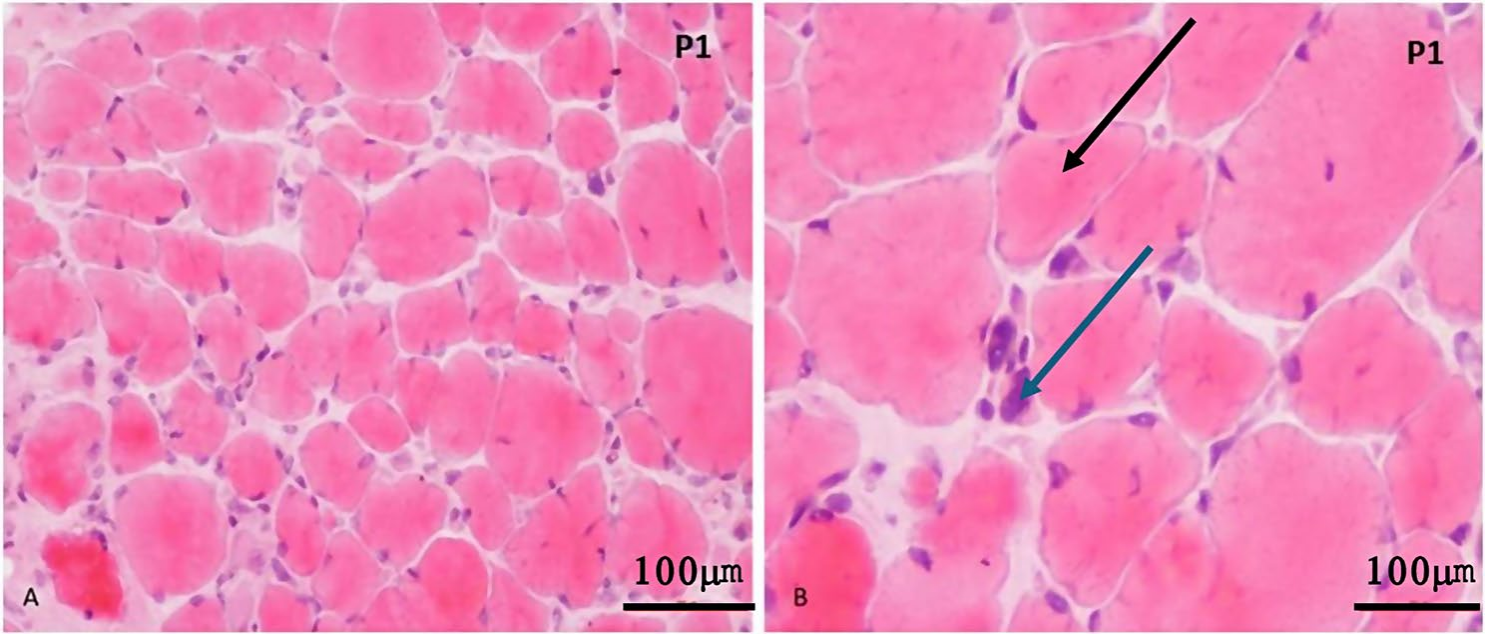
Photomicrograph showing variation in muscle fibre size, lobulated muscle fibres (black arrow), atrophic fibres (blue arrow) and internalization of nuclei (**a**: H and E x 20X, **b**: H and E x 40X)

The muscle biopsies on histology showed dystrophic features. The transverse muscle fibers revealed mild to moderate muscle fiber size variation. Many round atrophic and hypertrophic fibers were observed with internalization of nuclei (Fig. 1a). Lobulated (Fig. 1b) and split fibers were also noted. Occasional fibers showed subsarcolemmal reaction products but no ragged fibers were seen. There was no evidence of granuloma, vasculitis or amyloid deposition. Few necrotic fibers with focal myophagocytosis were observed. There was an increase in interstitial macrophages destroying muscle fibers. In most of the cases, mild inflammatory components were noted (supplementary Fig. 1).

### Enzyme histochemistry and immunohistochemistry

**Fig. 2:**
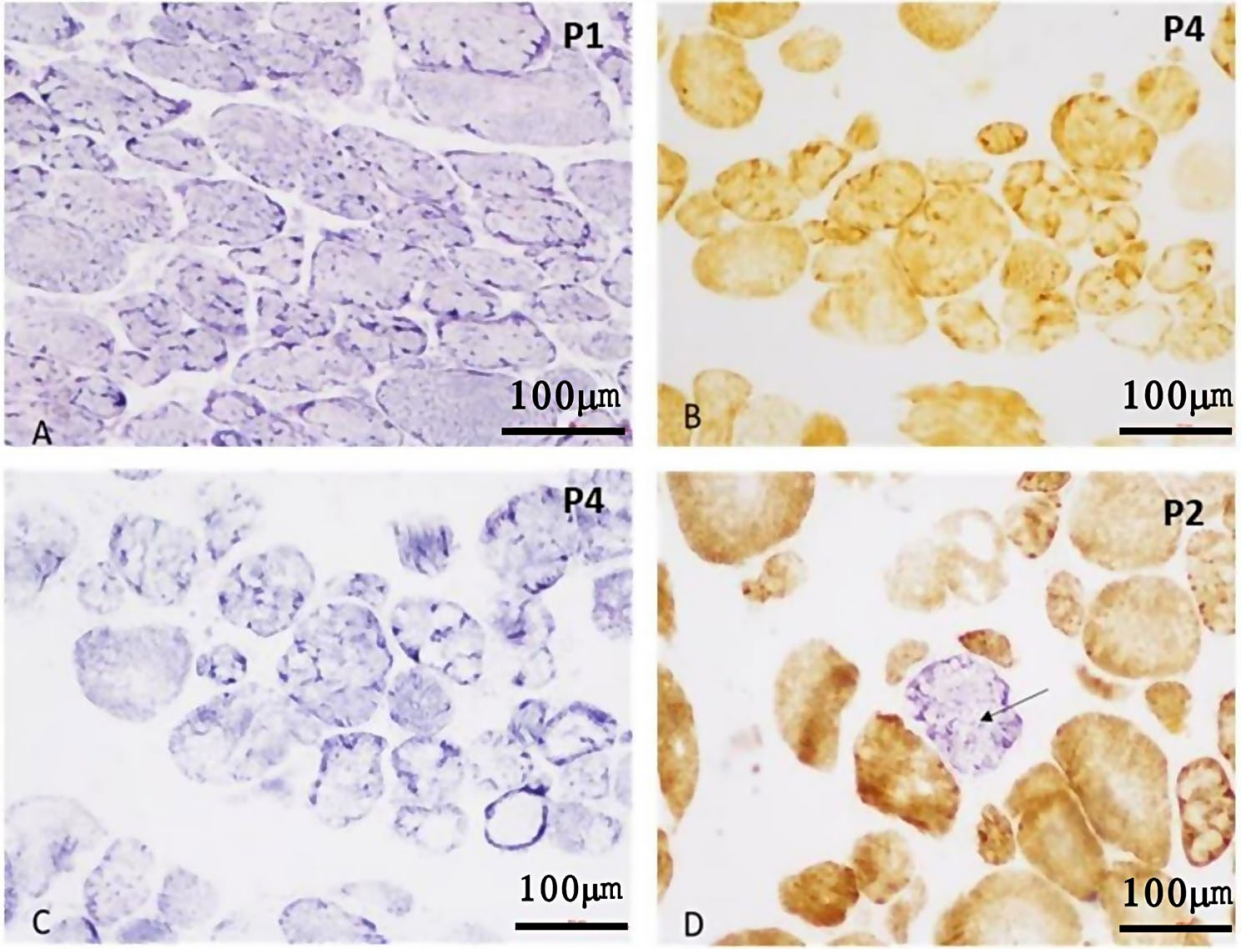
Enzyme histochemistry showing lobulated fibres **a**: NADH-TR, **b**: COX, **c**: SDH stains and *d*: combined COX-SDH for highlighting COX-deficient fibre (arrow) x 40X

Enzyme histochemistry was done using NADH-TR, COX, SDH, COX-SDH stains (Fig. 2a-d). NADH-TR stain highlighted the lobulated fibers. On NADH, the Mitochondria and sarcoplasm stained blue-purple due to formazan precipitate. Type I fibers (red slow oxidative) were darker than type II (white fast glycolytic). On COX stain, type I fibers were darker than type II. SDH stain was used to check mitochondrial proliferation and showed dark fibers rich in mitochondria. Combined COX-SDH stain showed fibre (blue) devoid of COX but expressing SDH.

**Fig. 3:**
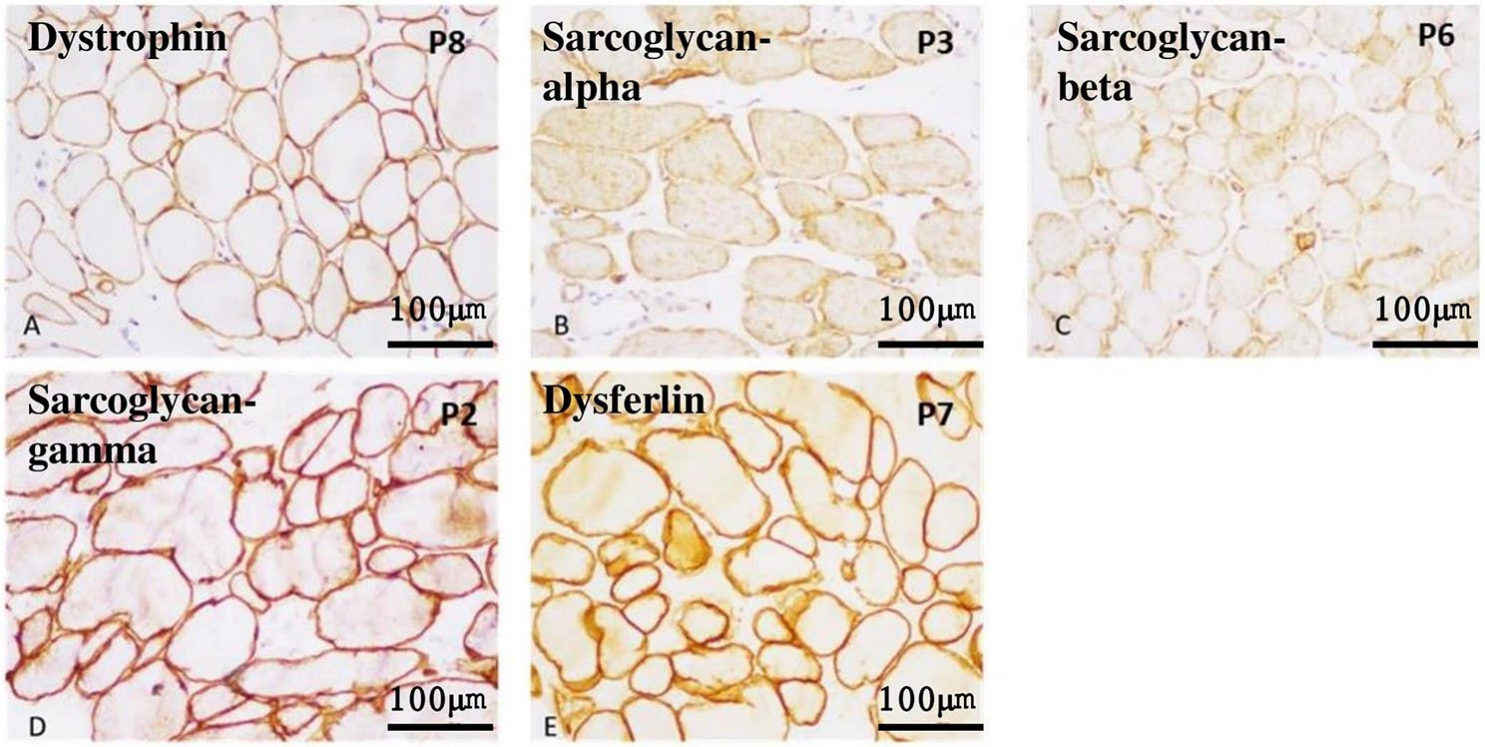
Immunohistochemistry using antibodies against Dystrophin, Sarcoglycan-alpha, Sarcoglycan-beta, Sarcoglycan-gamma and Dysferlin showing normal expression in myofibres (**a**: Dystrophin x 20X, **b**: Sarcoglycan-alpha x 40X, **c**: Sarcoglycan-beta x 20X, **d**: Sarcoglycan-gamma x 40X, e: Dysferlin x 40X)

Immunohistochemistry on frozen muscle sections using antibodies against dystrophin, sarcoglycan alpha, sarcoglycan beta, sarcoglycan gamma, dysferlin revealed normal expression on myofibers (Fig. 3a-e).

### Immunoblot analysis of Calpain-3 protein

**Fig. 4:**
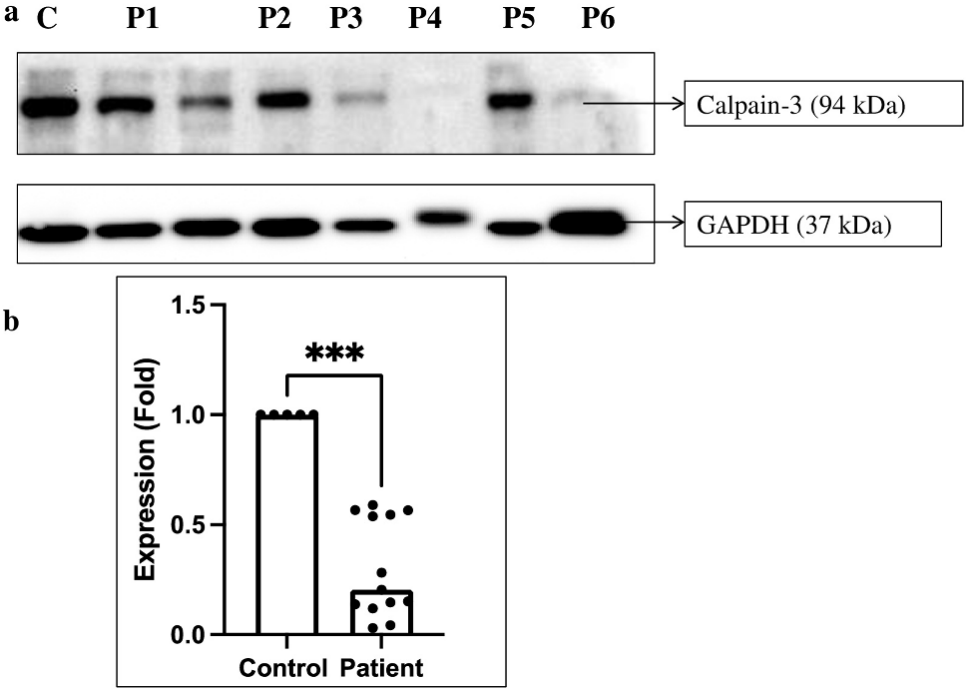
Immunoblot analysis of calpain-3 protein in LGMD patients (**a**) Decreased band intensity of calpain-3 protein at 94 kDa in patients as compared to control samples [The full image of membrane was not available. The membrane was cut and hybridised one part with anti-calpain-3 antibody and another part with anti-GAPDH antibody]; (**b**) Calpain-3 protein expression was significantly reduced in patients (13 LGMDR1 patients) as compared to control [C- Control; P- Patients 1–6]

A total 34 LGMD patients and 5 control subjects were subjected to immunoblot analysis of Calpain-3 protein. Out of 34 LGMD patients, 13 patients showed decreased expression of calpain-3 protein among which 2 patients showed complete loss of calpain-3 protein, whereas 11 patients showed partial loss of calpain-3 protein at its molecular weight location (94 kDa). As seen from Fig. 4a and supplementary Fig. 2, Patients (P) 1, 2, 3, 5 and 6 showed partial loss of Calpain 3, while patient 4 showed a total loss of the protein at 94 kDa position. GAPDH (37 kDa) was used as a loading control on the Western blot (Fig. 4a and supplementary Fig. 3). Calpain-3 protein expression was significantly reduced in 13 patients [*P* = 0.0002] as compared to controls (Fig. 4b).

### Sanger sequencing of *CAPN3* gene

**Fig. 5:**
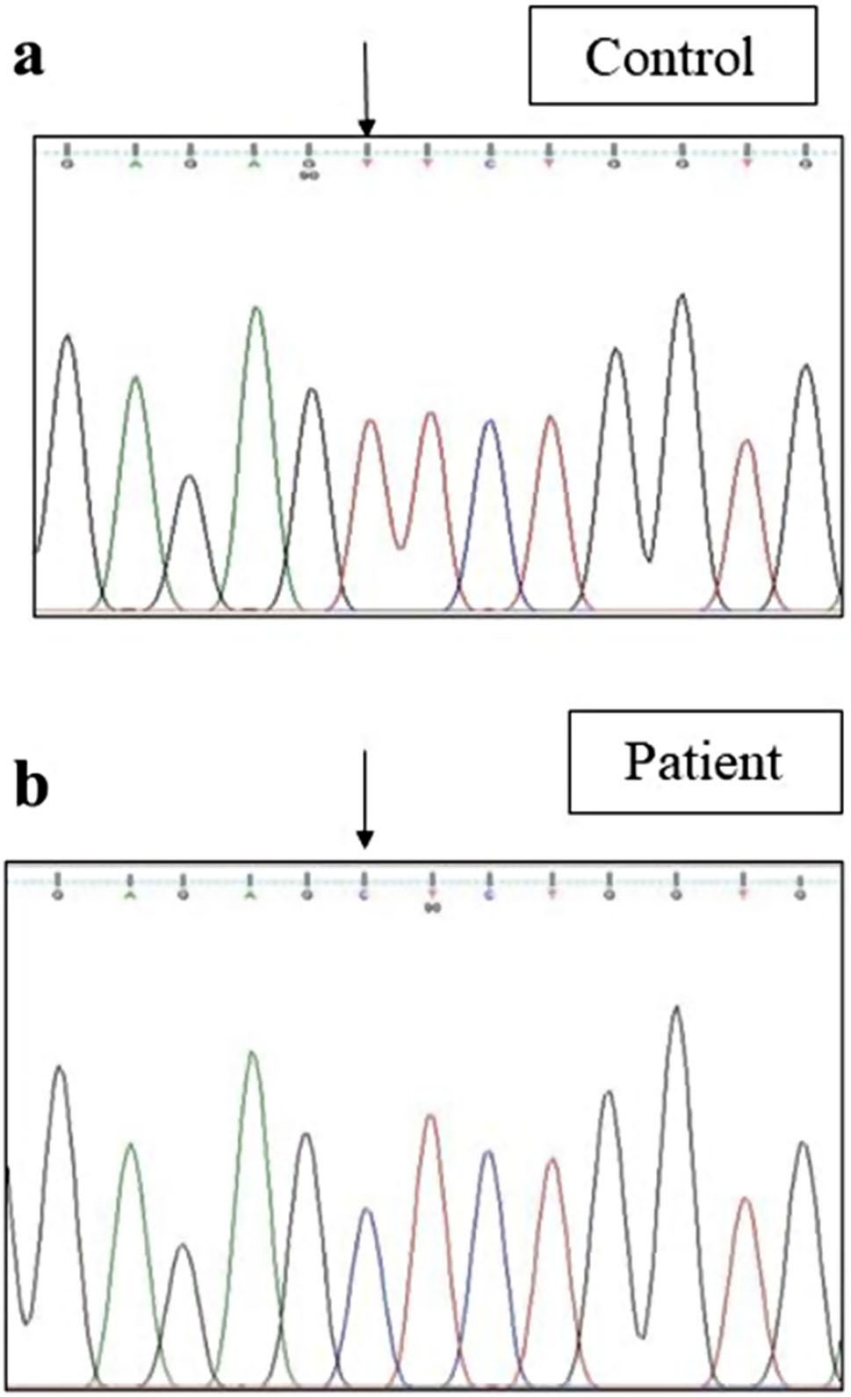
Homozygous missense variant in exon 9 of *CAPN3* gene (Patient 1) [(NM_000070.3 (*CAPN3*): c.1189T > C; NP_000061.1: p.Phe397Leu (Novel variant)] [**a** - control, **b** - patient]

**Fig. 6:**
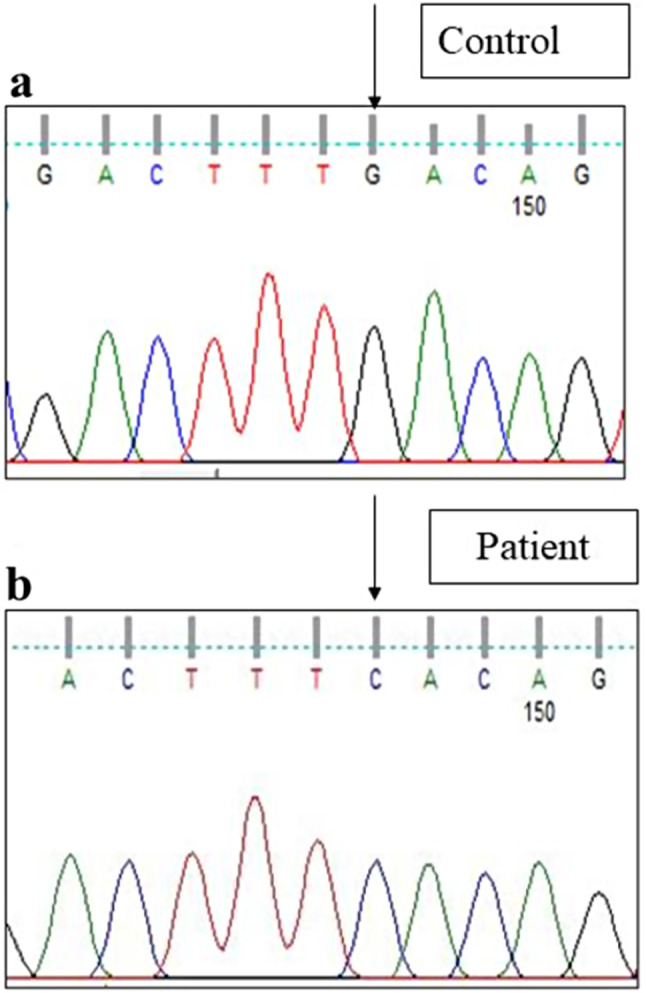
Homozygous missense variant in exon 22 of *CAPN3* gene (Patient 2) [NM_000070.3 (*CAPN3*): c.2338G > C; NP_000061.1: p.Asp780His (rs778768583)] [**a** - control, **b** - patient]

**Fig. 7:**
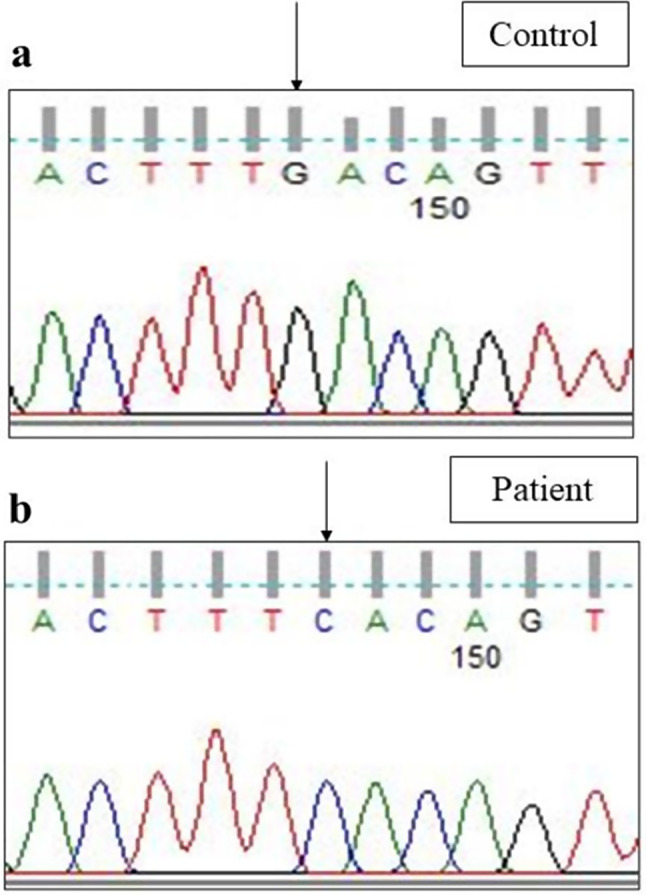
Homozygous missense variant in exon 22 of *CAPN3* gene (Patient 5) [NM_000070.3 (*CAPN3*): c.2338G > C; NP_000061.1: p.Asp780His (rs778768583)] [a - control, b– patient]

**Fig. 8:**
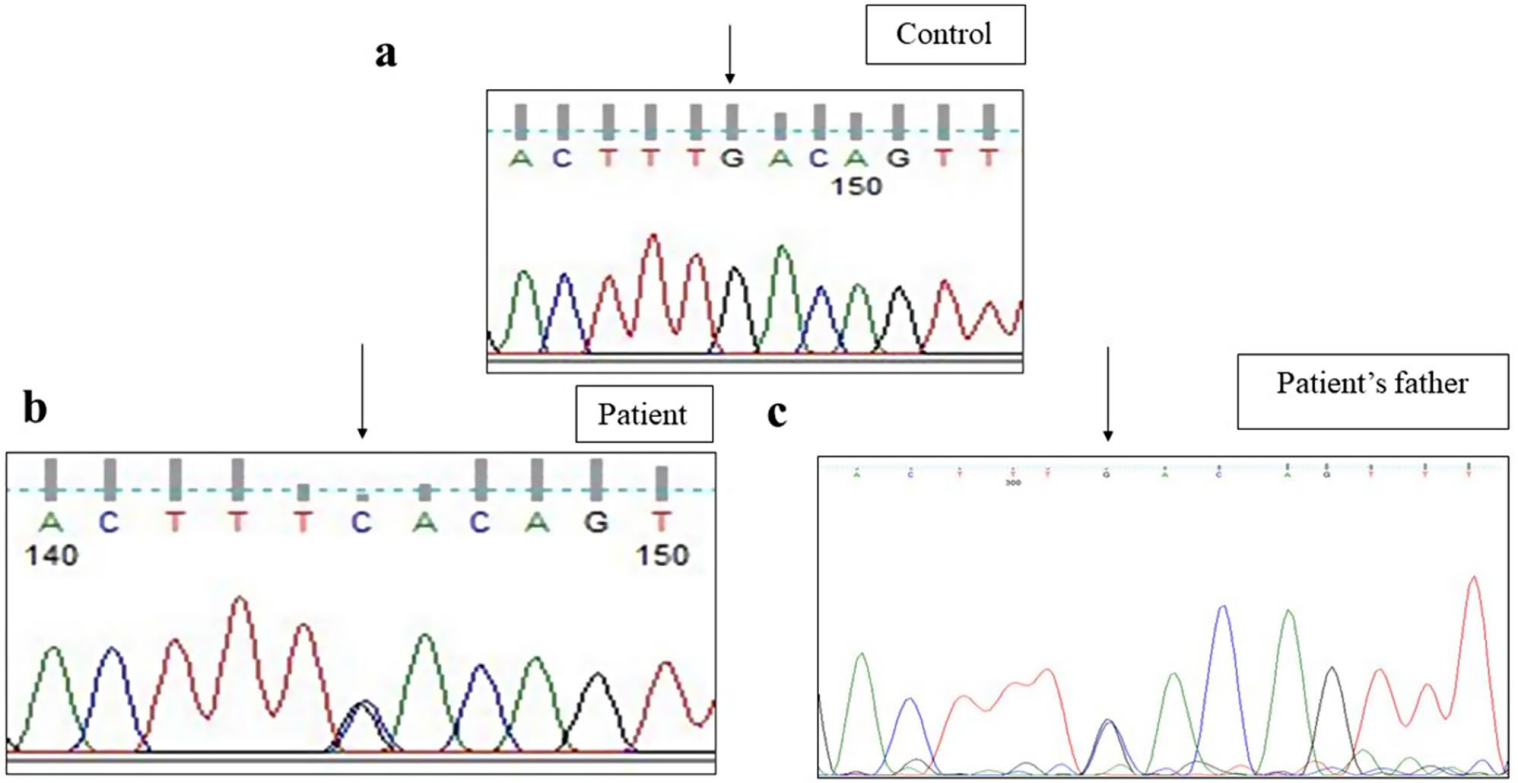
Heterozygous missense variant in exon 22 of *CAPN3* gene (Patient 3) [NM_000070.3 (*CAPN3*): c.2338G > C; NP_000061.1: p.Asp780His (rs778768583)] [**a** - control, **b**– patient, **c**- patient’s father]

**Fig. 9:**
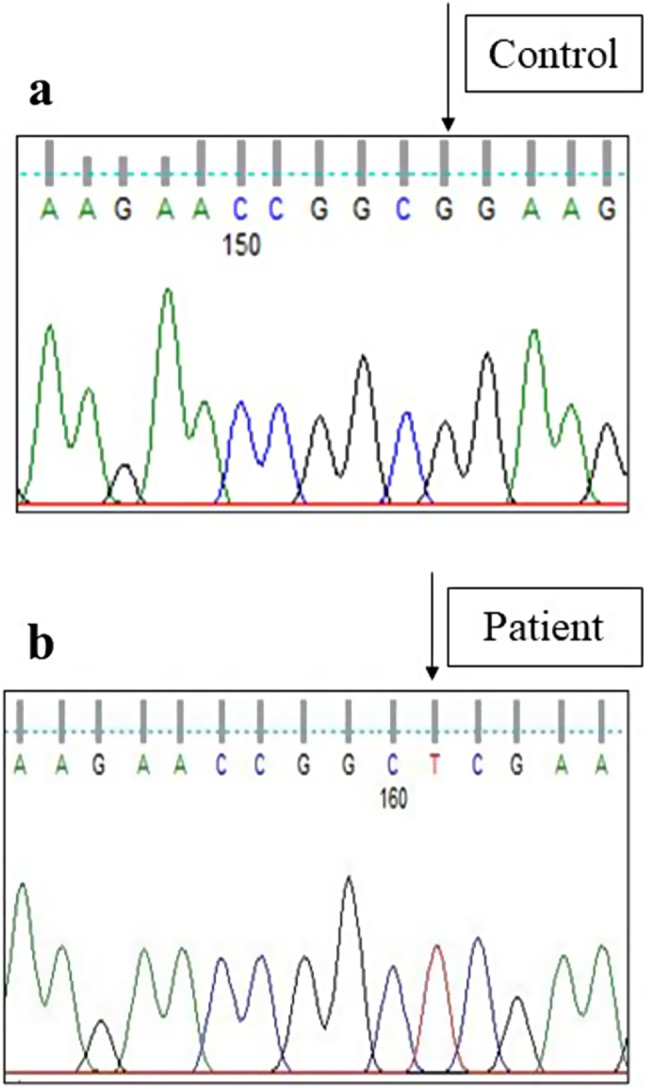
Homozygous insertion-deletion in exon 11 of *CAPN3* gene (Patient 4) [NM_000070.3 (*CAPN3*): c.1688delinsTC; NP_000061.1: p.Arg490Leufs*87 (Novel variant)] [**a** - control, **b** - patient]

**Fig. 10:**
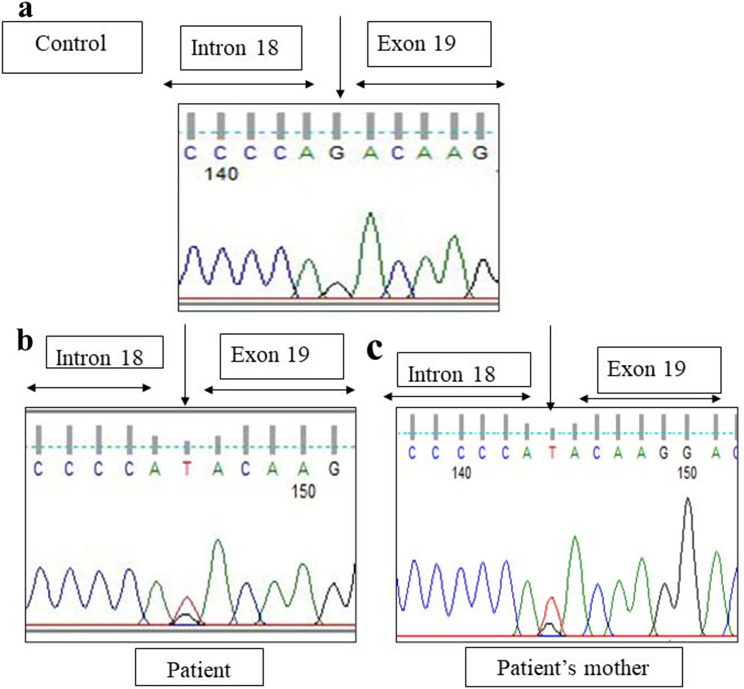
Splice site changes in intron 18 of *CAPN3* gene (Patient 3) [NM_000070.3 (*CAPN3*): (c.2051-1G > T) (rs886042108)] [**a** - control, **b**– patient, **c** - patient’s mother ]

**Fig. 11:**
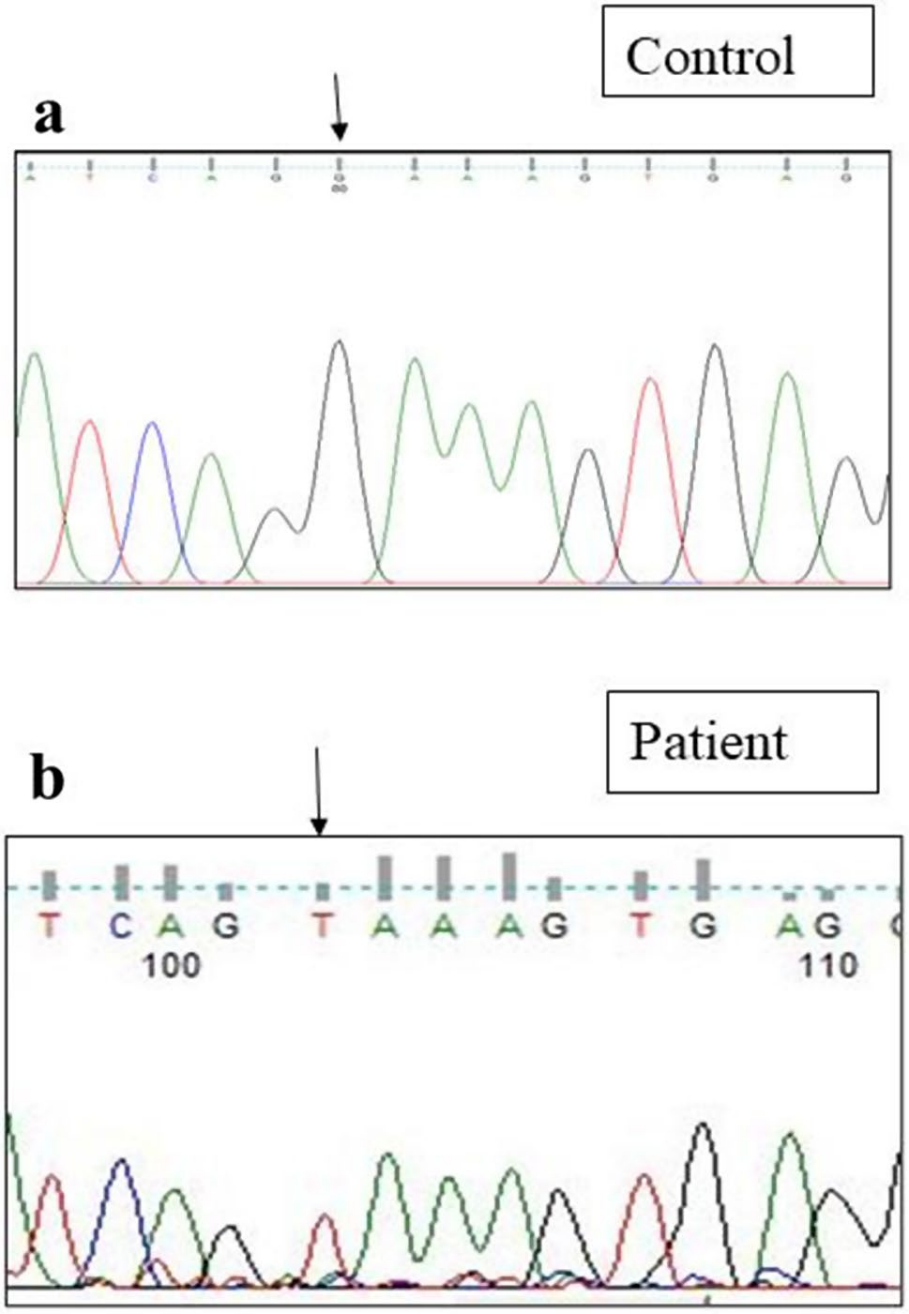
Homozygous nonsense variant in exon 17 of *CAPN3* gene (Patient 6) [NM_000070.3 (*CAPN3*): c.1939G > T; NP_000061.1: p.Glu647Ter **(**rs863224960)] [**a** - control, **b** - patient]

Sanger sequencing of total 24 exons of *CAPN3* gene was performed in all 34 LGMD patients. We found variants in *CAPN3* gene in 6 patients where calpain-3 protein expression was reduced. No *CAPN3* variants were identified in patients with normal expression of calpain-3 protein. Among all *CAPN3* variants in 6 LGMDR1 patients, two were missense [*CAPN3*: c.1189T > C (p.Phe397Leu), *CAPN3*: c.2338G > C (p.Asp780His)] (Figs. 5, 6, 7 and 8), one insertion-deletion [*CAPN3*: c.1688delinsTC (p.Arg490Leufs*87)] (Fig. 9), one splice site variant in intron 18 (c.2051-1G > T) (Fig. 10), one nonsense variant [*CAPN3*: c.1939G > T (p.Glu647Ter) (Fig. 11) (depicted in Table 1). Five patients had homozygous variants and one patient was compound heterozygote.

In summary, two novel variants - one missense, one insertion-deletion [*CAPN3*: c.1189T > C; *CAPN3*: c.1688delinsTC] in two patients while previously reported three variants- one missense, one nonsense, one splice-site [*CAPN3*: c.2338G > C; *CAPN3*: c.1939G > T; *CAPN3*: c.2051-1G > T] were observed in other 4 patients of LGMDR1 (depicted in Table 1). In our study, missense variants in exon 22 of *CAPN3* gene showed the highest frequency (3 out of total 6 patients = 50%) in LGMDR1 patients, all of which were predicted to be pathogenic.

## Discussion

LGMD is the most frequent form of muscular dystrophy worldwide [13] and accounts for around 40% of all LGMD cases based on different regions [27]. The current investigation results account for 38.2% of total LGMD cases as calpainopathy is similar to these studies. The study was conducted to identify novel variants in *CAPN3* gene in LGMDR1 patients. A total of thirty-four cases with dystrophic features on histology and suspected to have LGMD were enrolled in the study. Immunoblot analysis was done in 34 LGMD cases to detect expression of calpain-3 protein in muscle biopsies among which 13 patients showed reduced expression. Significant reduction of calpain-3 protein (94 kDa) on Western blot was observed in these 13 patients (6 patients with *CAPN3* variants and 7 without *CAPN3* variants) as compared to controls. Our observation was similar to the previous study where reduced or absent expression of calpain-3 protein was observed at 94 kDa band size [28].

Secondary reduction of calpain-3 protein can be observed in other types of muscular dystrophies. Thus, immunohistochemistry of other muscle proteins should be done to exclude other type of muscular dystrophies for patients with clinical features of LGMDR1 [8]. In our study, muscle biopsies showing normal protein expression of dystrophin, dysferlin, sarcoglycans were selected for immunoblot. Calpainopathy is considered as a group of myopathies with various phenotypes [29]. In LGMDR1, CK level increases at onset and decreases during progression however may remain elevated at all stages [25]. In our study, CK level was found to be increased in LGMDR1 patients. Sometimes calpainopathy patients may show normal protein expression with variation in *CAPN3* gene [30]. Therefore, genetic analysis of *CAPN3* gene is required after immunoblot analysis of calpain-3 protein in these patients.

### Novel variants

LGMDs are characterized as a heterogeneous group of neuromuscular disorders with proximal muscle weakness regarding the range of age of onset, clinical symptoms, genotype, and disease duration [17]. In this present study, we found two novel variants among which one was missense variant and other was insertion-deletion variant. In patient 1, missense likely pathogenic variant c.1189T > C (p.Phe397Leu) in exon 9 triggered alteration in amino acid change phenylalanine (nonpolar aromatic amino acid) to leucine (nonpolar aliphatic amino acid) (Fig. 5b) compared to control (Fig. 5a). In-silico analysis (variant prediction tool-mutation taster) showed that catalytic site of calpain-3 protein may be lost in this case. This novel missense variant was classified as likely pathogenic according to ACMG guidelines. Out of 2 novel variants, we also found one insertion-deletion variant leading to frameshift. Novel insertion-deletion variant c.1688delinsTC (p.Arg490Leufs*87) was observed in exon 11 in another patient (Patient 4), where G was deleted at 1688 position and TC was inserted, causing alteration of arginine to leucine resulting in premature termination of protein formation (Fig. 9b) compared to control (Fig. 9a). In-silico analysis (variant prediction tool- mutation taster) of this variant showed that such insertion and deletion could cause loss of domain III (a calcium and phospholipid binding C2 domain) of calpain-3 protein.

### Reported variants

We found three previously reported pathogenic variants in LGMDR1 patients among which one was missense variant, one was splice site variant, and another one was nonsense variant. In this study, we found a previously reported (rs778768583) [31–33] missense pathogenic variant (c.2338G > C) (p.Asp780His) in exon 22 (Figs. 6b, 7b and 8b) compared to controls (Figs. 6a, 7a and 8a), in 3 patients, resulting in change of aspartic acid to histidine. In our study, parents of LGMDR1 also showed the same variants observed in their offsprings but were asymptomatic. In patient 3, missense variant (c.2338G > C) was seen in exon 22; in this case father was heterozygous (Fig. 8c). This variant has been established as founder mutations for the Agarwal community in India [32]. In-silico analysis of this variant using the variant prediction tool- mutation taster, predicted that in such a variant, the domain IV (calmodulin-like Ca2 + binding domain containing 5 EF hand sequences) of calpain-3 protein was affected, which could be responsible for the pathogenicity. In one case (patient3), this mutation was accompanied by another mutation, a previously reported [32] splice site variant (c.2051-1G > T) (rs886042108) in intron 18 of *CAPN3* gene (Fig. 10b) compared to control (Fig. 10a), which was predicted to be pathogenic. The mother of this same patient (patient 3) was found to be heterozygous (Fig. 10c). Apart from this, a homozygous nonsense pathogenic variant c.1939G > T (p.Glu647Ter) **(**rs863224960**)** was found in exon 17 of *CAPN3* gene (Fig. 11b) [34, 35] in patient 6 compared to control (Fig. 11a), resulting in termination of protein formation at 647 position after formation of a premature stop codon. In-silico analysis (variant prediction tool- mutation taster) of this variant showed that the linker region of *CAPN3* gene was lost.

### Genotype-phenotype correlation

Genotype-phenotype correlation is the association between specific mutations and the spectrum of disease expression. Proximal muscle weakness was observed in all 6 patients as the main clinical manifestation. Genotype-phenotype correlation of LGMDR1 patients with novel and reported variants are discussed below.

LGMDR1 patients with novel variants were diagnosed with proximal muscle weakness and symptomatic since last 3–10 years. One out of two patients (except patient 4) had scapular winging as main clinical feature. Patient (Patient 1) with homozygous likely pathogenic missense variant in exon 9 [*CAPN3*: c.1189T > C (p.Phe397Leu)] had bilateral upper limb since last 2 years, difficulty in getting up from squatting position and climbing upstairs as clinical symptoms. This patient had a history of consanguinity. This missense variant in exon 9 of patient 1 may affect calpain-3 protein expression, which was reduced on immunoblot analysis. Patient (Patient 4) with homozygous insertion-deletion in exon 11 [*CAPN3*: c.1688delinsTC (p.Arg490Leufs*87)] had difficulty in walking and required walking stick. The insertion-deletion variant in patient 5 may affect calpain-3 protein expression which was absent on immunoblot analysis.

The missense variant in exon 22 may affect calpain-3 protein expressions in patient 2 and patient 5 which were found to be reduced on immunoblot analysis. Patient (Patient 3) was found to be compound heterozygote with splice site variant in intron 18 [*CAPN3*: c.2051-1G > T] and heterozygous missense variant in exon 22 [*CAPN3*: c.2338G > C (p.Asp780His)]. The acceptor splice site variant at the boundary of intron 18-exon 19, may disrupt RNA splicing which results into severely reduced calpain-3 protein expression observed in that particular patient (patient 3). The nonsense variant found in exon 17 of patient 6 may affect calpain-3 protein which was severely reduced on Western blot.

In our study, the correlation between genotype and phenotype indicated physical traits in LGMDR1 patients with a given pathogenic genetic variant. Patients with nonsense and insertion-deletion (indel) variants were associated with severe diseased conditions and wheelchair-bound in twenties. In one patient (patient 4) with homozygous indel variant leading to frameshift, calpain-3 protein was totally absent. This observation supports previous studies where it is mentioned that null variants cause mostly total loss of calpain-3 protein [11, 35]. We observed comparatively less severe phenotype in patients with missense *CAPN3* variants which is in concordance with previous studies due to variability of their effect at protein level [9, 16, 36]. However, this genotype-phenotype correlation needs to be studied in a larger number of LGMDR1 patients.

### Limitation

There were some limitations in our study. For Western blotting, we used Abcam anti-calpain-3 antibody (ab103250) which detected calpain-3 protein only at the 94 kDa band position (Supplementary Fig. 4). Therefore, we considered the band reduced or absent for calpain-3 protein, corresponding to the size of 94 kDa for the diagnosis of calpainopathy. Previous studies used another antibody i.e. Novocastra anti-calpain-3 antibody (NCL-CALP-12A2) to detect calpain-3 protein expression. This antibody was not available during the study period which detects other fragments of calpain-3 protein i.e. 60 kDa and 55 kDa along with 94 kDa [12]. The study by Pathak P et al., 2010 reported that expression of other fragments of calpain-3 protein (60, 58, and 55 kDa) was reduced in LGMDR1 patients compared to control using NCL-CALP-12A2 antibody [25]. Their observation was also similar to other previous studies by Fanin M et al., 2009, Fanin M et al., 2001 [28, 35]. In another study by Luo et al., 2011, only 94-kDa band and 60-kDa band were considered to detect calpain-3 protein expression by Western blotting using NCL-CALP-12A2 antibody [29]. Additionally, we could have performed titin immunohistochemistry (IHC) as calpain-3 reduction on Western blot can be secondary to other muscle-related protein deficiencies [37]. Out of 34 LGMD patients, 13 patients had reduced calpain-3 protein expression by immunoblot analysis. *CAPN3* variants were found in 6 out of theses 13 patients while *CAPN3* variants were absent in remaining 7 patients. Previous study by Bevilacqua JA et al. 2020 reported that the nine most-frequent LGMD-causing genes were CAPN3; DYSF; SGCG; SGCA; SGCB; SGCD; FKRP; ANO5; TCAP and GAA [38]. Thus, high throughput genome wide sequencing could be done in these 7 patients (without *CAPN3* variants) to detect presence of other variants in the above-mentioned LGMD genes. Therefore, we have mentioned these 7 patients (without *CAPN3* variants) as suspected LGMDR1 in the limitation part of our study.

## Conclusion

Identification of novel variants in *CAPN3* gene in our study may help to find out the genetic cause of LGMD in patients suffering from the disease right from childhood to adulthood. Confirmation of diagnosis may avoid unnecessary treatments given to such patients and it may also help in genetic counseling as well as to formulate therapeutic interventions. Genetic factors may regulate the diseased phenotype of LGMDR1; therefore, genetic testing can help to understand the molecular basis of pathogenesis of these heterogeneous patients. Thus, genotype-phenotype correlations are required to elucidate characteristics of a mutation and its effect on a patient’s phenotype with a genetic etiology. However, such studies involving mutation analysis of the *CAPN3* gene should be carried out only after conducting immunoblot analysis of the protein, which helps in diagnosis of LGMDR1, in conjunction with clinical, pathological and immunohistochemical findings. Although in most cases, genetic studies are sufficient to diagnose LGMDR1 which may avoid the need of muscle biopsy.

### Electronic supplementary material

Below is the link to the electronic supplementary material.

Supplementary Material 1

Supplementary Material 2

Supplementary Material 3

Supplementary Material 4

## Data Availability

The data that support the findings of this study are not openly available due to reasons of sensitivity and are available from the corresponding author upon reasonable request.

## Declarations

(b) All procedures performed in studies involving human participants were in accordance with the ethical standards of the institutional and/or national research committee and with the 1964 Helsinki Declaration and its later amendments or comparable ethical standards.

## Abbreviations

*CAPN3*: Calpain-3
CK: Creatine kinase
COX-SDH: Cytochrome oxidase-succinic dehydrogenase
GAPDH: Glyceraldehyde 3-phosphate dehydrogenase
IHC: Immunohistochemistry
LGMDR1: Limb girdle muscular dystrophy recessive 1
NADH-TR: Nicotinamide adenine dinucleotide-tetrazolium reductase
