# Supplementary figures and images for: Identification of novel pathogenic variants of Calpain-3 gene in limb girdle muscular dystrophy R1
Source: Orphanet J Rare Dis. 2024 Apr 1;19:140. doi: 10.1186/s13023-024-03158-1 (PMC10983654; doi:10.1186/s13023-024-03158-1)

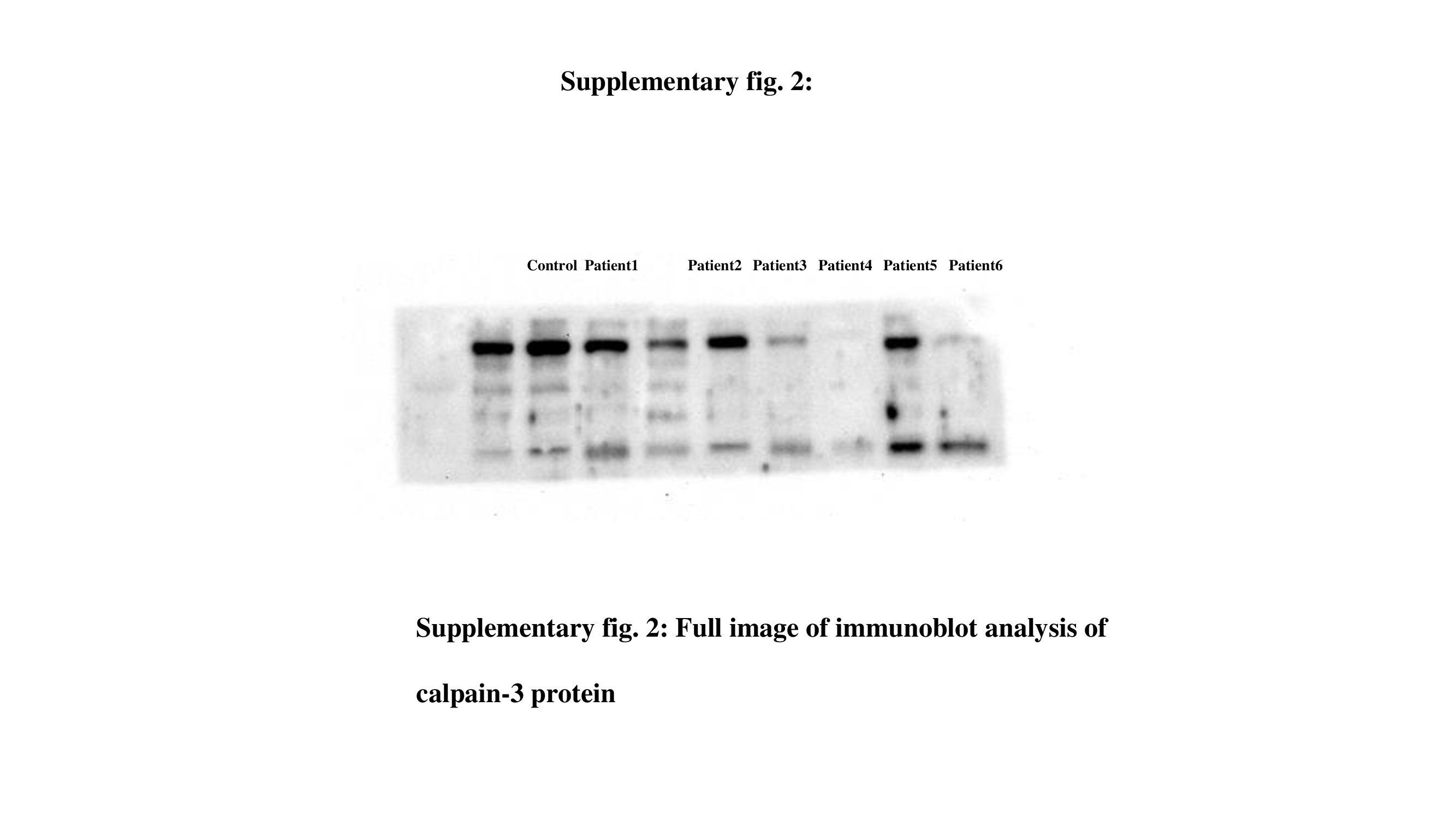

Supplement: Supplementary file 1 — Supplementary Material 1 [file 13023_2024_3158_MOESM1_ESM.jpg]

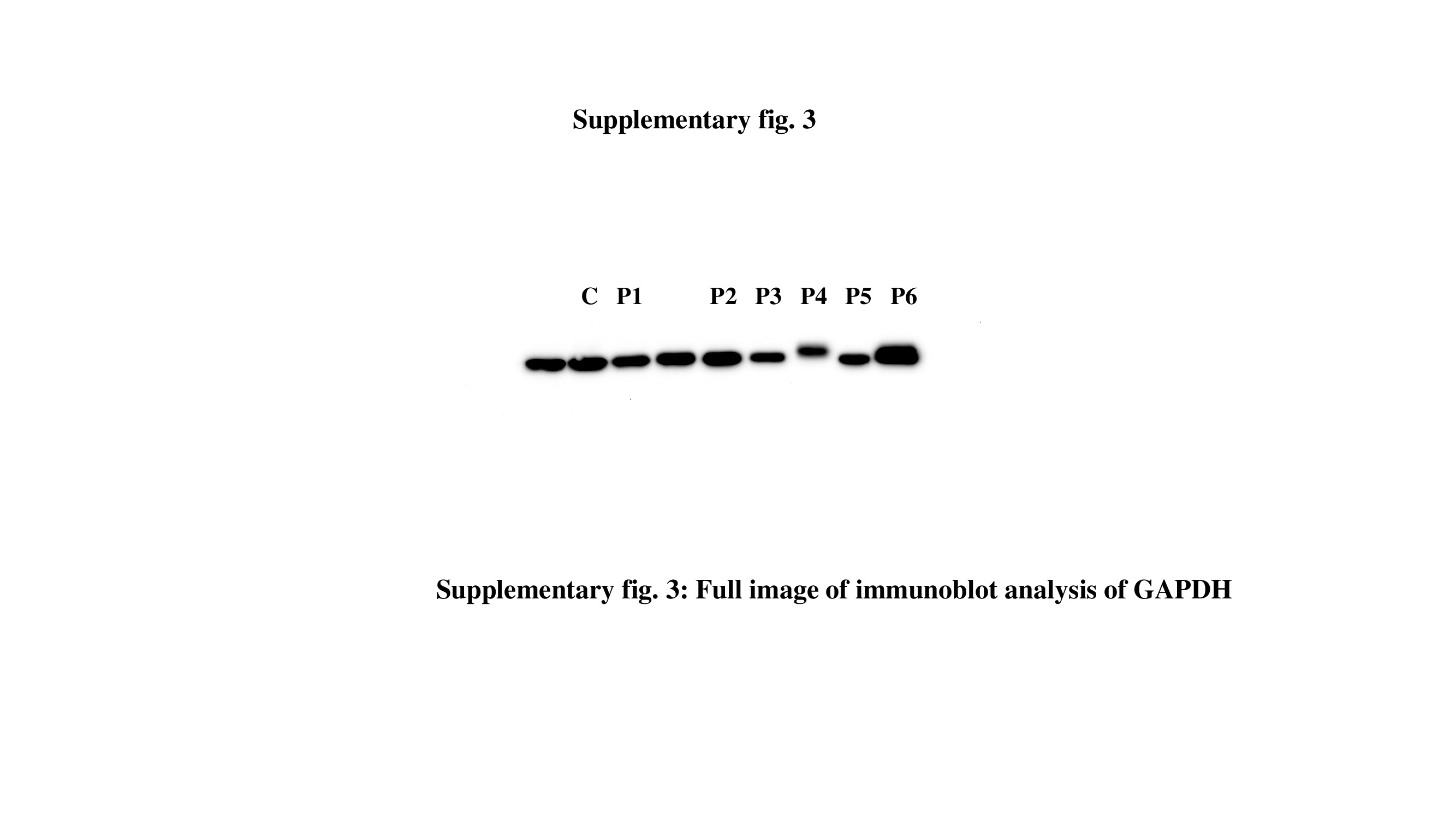

Supplement: Supplementary file 2 — Supplementary Material 2 [file 13023_2024_3158_MOESM2_ESM.jpg]

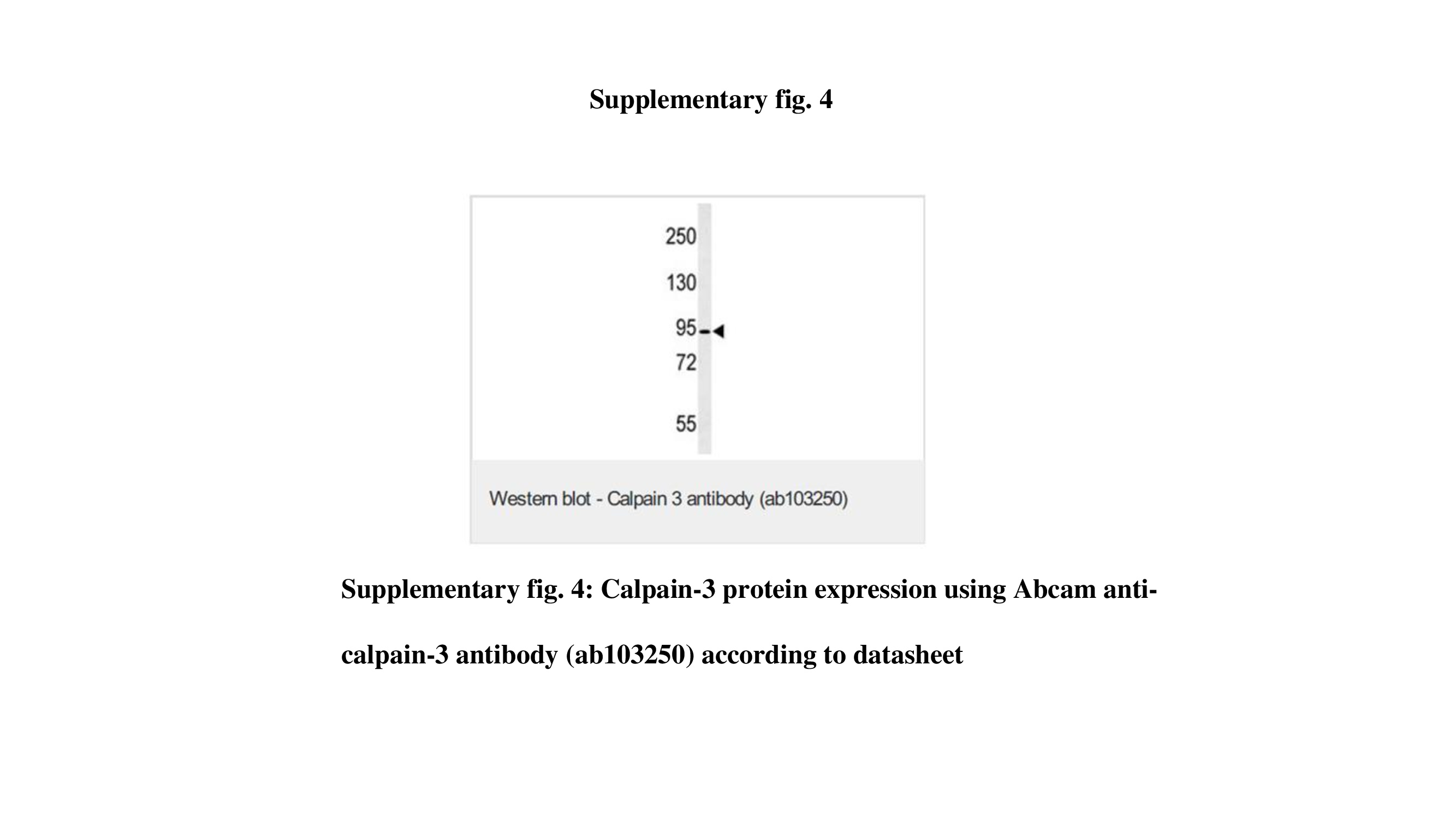

Supplement: Supplementary file 3 — Supplementary Material 3 [file 13023_2024_3158_MOESM3_ESM.jpg]

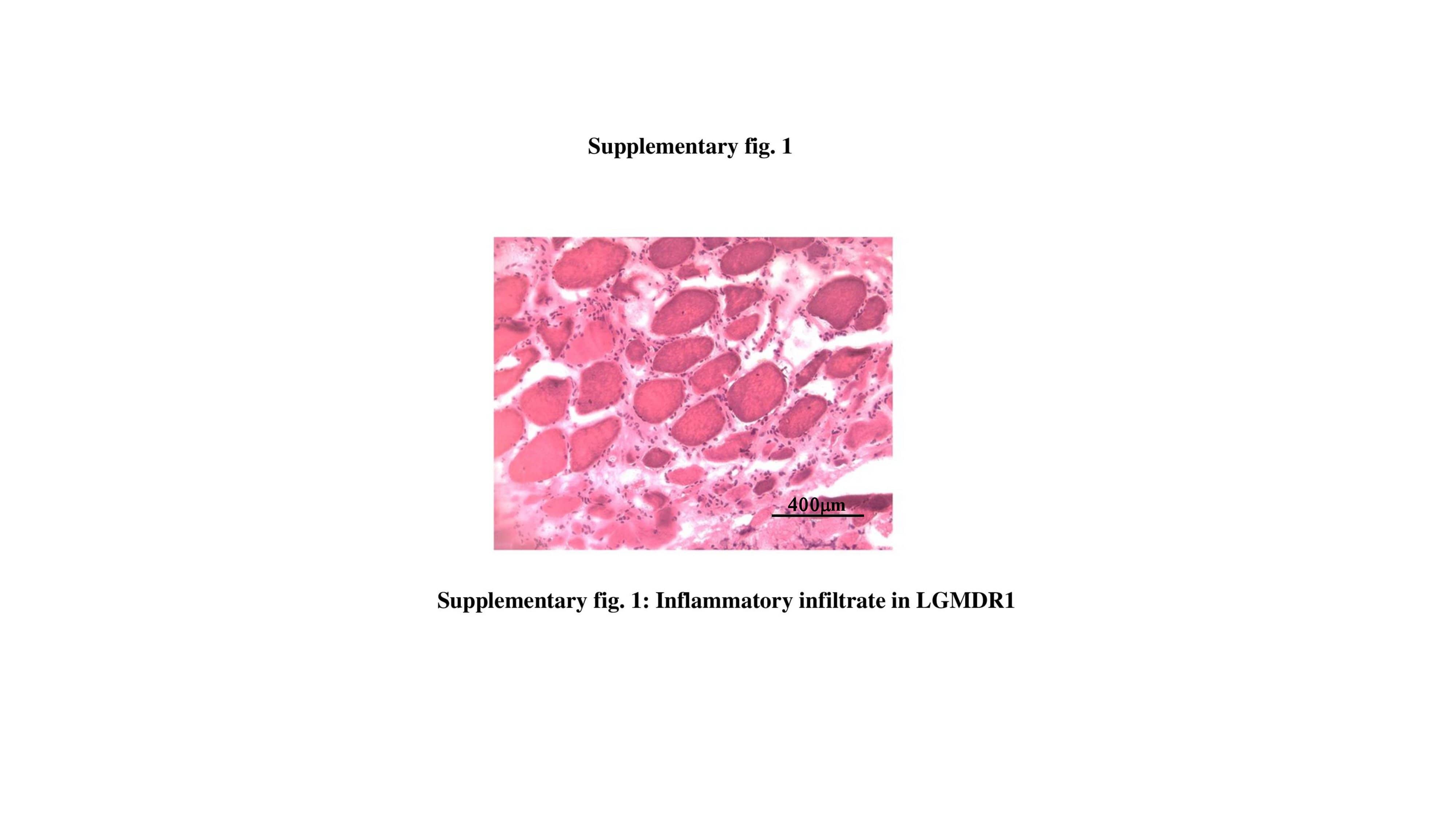

Supplement: Supplementary file 4 — Supplementary Material 4 [file 13023_2024_3158_MOESM4_ESM.jpg]
